# Influence of Gender and Undergraduate Course on the Knowledge about HPV and HPV Vaccine, and Vaccination Rate among Students of a Public University

**DOI:** 10.1055/s-0040-1701466

**Published:** 2020-02

**Authors:** Marília Biselli-Monteiro, Amanda Canato Ferracini, Luis Otávio Sarian, Sophie Françoise Mauricette Derchain

**Affiliations:** 1 Department of Obstetrics and Gynecology, Medical School, Universidade Estadual de Campinas, Campinas, SP, Brazil

**Keywords:** human papillomavirus, vaccine, students, knowledge, vaccination rate, vírus do papiloma humano, vacina, estudantes, conhecimento, taxa de vacinação

## Abstract

**Objective** To evaluate the knowledge related to human papillomavirus (HPV) infection and the rate of HPV vaccination among undergraduate freshmen and senior students of medicine, pharmacy, speech therapy, nursing and physical education in a Brazilian university.

**Methods** A questionnaire concerning sociodemographic aspects, sexual background, and knowledge about HPV and its vaccine was filled out by 492 students. Three months later, a second questionnaire, concerning the new rate of vaccination, was applied to 233 students.

**Results** Among the 290 women who answered the first questionnaire, 47% of the freshmen and 13% of the seniors stated they were not sexually active, as well as 11% of the 202 freshman and senior male students. Although the knowledge about HPV was higher among women, they reported a lower use of condoms. More than 83% of the women and 66% of the men knew that HPV can cause cervical cancer, but less than 30% of the students knew that HPV can cause vulvar, anal, penile and oropharyngeal cancer. Less than half of the students knew that HPV causes genital, anal and oropharyngeal warts. Comparing the students, the seniors had more knowledge of the fact that HPV is sexually transmitted, and that HPV infection can be asymptomatic. The rate of vaccination was of 26% for women, and of 8% for men, and it increased to 52% and 27% respectively among the 233 students evaluated in the second questionnaire.

**Conclusion** As almost half of freshman women declared being sexually inactive, the investment in public health information programs and easier access to the HPV vaccine seem to be a useful strategy for undergraduate students.

## Resumo

**Objetivo** Avaliar o conhecimento sobre a infecção pelo vírus do papiloma humano (*human papillomavirus*, HPV, em inglês) e a taxa de vacinação entre estudantes calouros e veteranos do quarto ano dos cursos de medicina, farmácia, fonoaudiologia, enfermagem e educação física de uma universidade brasileira.

**Métodos** Um primeiro questionário sobre aspectos sociodemográficos, antecedentes sexuais e conhecimento sobre o HPV e sua vacina foi aplicado a 492 estudantes. Três meses depois, foi aplicado um novo questionário, a 233 estudantes, que avaliava a nova taxa de vacinação entre eles.

**Resultados** Entre as 290 mulheres que responderam ao questionário, 47% das calouras e 13% das veteranas negaram início de atividade sexual. Entre os 202 calouros e veteranos do sexo masculino avaliados, essa taxa foi de 11%. Apesar de o conhecimento sobre o HPV ter sido maior entre as mulheres, elas declararam menor uso de preservativo. Mais de 83% das mulheres e 66% dos homens sabiam que o HPV causa câncer de colo de útero, mas menos de 30% de todos os alunos sabiam que o HPV pode causar câncer de vulva, ânus, pênis e orofaringe, e menos de 50% sabiam que o HPV pode causar verrugas genitais, anais e orofaríngeas. Comparando calouros e veteranos, houve um aumento no conhecimento de que o HPV é sexualmente transmitido, e de que sua infecção pode ser assintomática, entre os veteranos em comparação com os calouros. Pela taxa de vacinação analisada no segundo questionário, identificou-se que, antes do início da pesquisa, 26% das mulheres e 8% dos homens haviam sido vacinados, e, no momento da aplicação do segundo questionário, essas taxas subiram para 52% e 27%, respectivamente, entre os 233 alunos avaliados.

**Conclusão** Quase metade das calouras relataram não ser sexualmente ativas, e a maioria delas ainda não era vacinada contra o HPV. O ingresso no Ensino Superior parece um momento oportuno para a realização de campanhas governamentais de conscientização e vacinação contra o HPV.

## Introduction

Current academic knowledge pertaining to human papillomavirus (HPV) infection and its relationship with human pathology is vast and well established. Decades of cumulative investigation led to a firm amalgam of information concerning the biological basis for the different effects of HPV infection on the cervix, vulva, penis, anus and oropharynx.^1^ In essence, HPV is the major causal factor for genital warts and multiple cancers such as cervical, vulvar, vaginal, oropharyngeal, penile and anal cancer.^2^ Cervical cancer is the third most prevalent neoplasm and the fourth leading cause of death by cancer among women.^3^ The prevention of HPV infection can be accomplished through prophylactic vaccines, which, administered prior to the contact with the virus, provide almost 100% of efficacy. The vaccines are prophylactic, and have their greatest benefit in boys and girls who have not yet started their sexual activity. Sexually active adolescents and young adults are also vaccinated to catch up.^2^ The first vaccine against HPV has been approved by the US Food and Drug Administration (FDA) in 2006; however, the global vaccination rate for girls aged 10 to 20 years by 2015 was of 6.1%.^4^

In spite of the unequivocal progress obtained in the diagnosis, treatment and prevention of HPV infection, several studies suggest that the awareness and knowledge about the infection, its causes and risk factors among the population is surprisingly limited. Alarmingly, it is known that higher knowledge about HPV and its consequences is related to a higher propensity to take the vaccine,^5^ and ignorance on the part of the general population about the implications of HPV infection to general health is highly associated with the failure of prevention initiatives. McCusker et al^6^ applied a questionnaire concerning knowledge about HPV to freshman medicine students at a university in Scotland. The students participated later in a public health information (PHI) campaign that described the role of HPV in the development of cervical cancer. After the PHI, they observed a significant increase in the rate of HPV vaccination among girls: in 2008, before the campaign, no girl had taken all three doses of the vaccine; however, one year after the intervention, more than 58% were vaccinated. They also reported that after this campaign, 94% of the vaccinated girls understood that they should remain in the screening program.^6^

It is therefore clear that awareness about HPV infection and its consequences among the general population is surprisingly low considering the consolidated academic knowledge about it, its clinical implications, and the ample amount of diagnostic and therapeutic tools currently available. Even more alarming, a few pioneering studies suggest that in the developed and developing world, young adults studying in healthcare-related undergraduate programs may display unacceptable low levels of knowledge about HPV infections as well. Our hypothesis is that freshman students go to college between the ages of 18 and 25 years, and, therefore, are at a high risk for HPV infection, but only a small proportion of those students are vaccinated against HPV. Our objective was to perform a comprehensive longitudinal evaluation of the knowledge related to HPV and HPV vaccination, among freshman and senior undergraduate students of medicine, pharmacy, speech therapy, nursing and physical education in an extremely competitive Brazilian university. We also evaluated the proportion of students already vaccinated before the questionnaire, and the proportion of those who took the vaccine within three months of the application of the questionnaire.

## Methods

The present was an observational cohort study. After approval by the Ethics in Research Committee of Universidade Estadual de Campinas (Unicamp, in Portuguese) (under CAAE 64275917.4.0000.5404), a questionnaire was applied in August 2017 to freshman and senior undergraduate students aged ≥ 18 years of the courses of medicine, nursing, speech therapy, pharmacy and physical education of Unicamp. All subjects signed the free and informed consent form. The questionnaire was composed of 79 multiple-choice, true or false, and short answers; it included sociodemographic aspects, sexual background, knowledge on HPV and the vaccine, and the rates of vaccination and of intention to indicate the vaccine to girls and boys in their professional future. After the students filled out the questionnaire, a sheet with the correct answers was offered to them. A second questionnaire with 5 questions was applied in November 2017 to 233 students, with the objective of analyzing the vaccination rate of the medicine and pharmacy undergraduates, and to evaluate if those who had participated in the first part of the survey were more likely to get vaccinated. The transcription of the questionnaire data was performed using the REDCap (Vanderbilt University, Nashville, TN, US) web application. The data was then exported and analyzed using the R Environment for Statistical Computing (R Foundation for Statistical Computing, Vienna, Austria).^7^ The Chi-squared test for trends and the *t*-test were used to evaluate differences in knowledge across the groups of students, as well as the changes in the vaccination rate before and after the application of the questionnaires. Due to the small number of students and the similarity in the sociodemographic characteristics, the data obtained from the speech therapy and nursing students were grouped.

## Results

### Sociodemografic Factors

Among the 492 students included in the present study, there were 290 women and 202 men. In total, there were 196 medicine students, 63 pharmacy students, 39 speech therapy students, 44 nursing students, and 147 physical education students. There were 279 (56.7%) freshmen and 213 (43.3%) seniors. The proportion of female students was significantly higher in the nursing (97.7%) and speech therapy (94.9%) courses compared with pharmacy (79.7%), medicine (57.4%) and physical education (31.3%) (*p* < 0.001). A significantly larger proportion of medical students reported living alone, in boarding homes or with friends, compared with those of other courses, who most often reported living with their parents. The rate of students who declared themselves as followers of a religion was higher among the nursing and speech therapy students when compared with those of other courses (*p* < 0.05). Most students reported having graduated from high school in a public or technical school, excluding the pharmacy students. The proportion of students who had at least one parent that was a college graduate was significantly higher among the pharmacy (74.2%) and medicine (77.6%) students compared with the physical education (61%) and nursing/speech therapy (50%) students (*p* < 0.01) (Table 1).

**Table 1:** Sociodemographic characteristics of the study sample

Sociodemographic characteristics	Physical education n(%)	Speech therapy n(%)	Nursing n(%)	Nursing and speech therapy n(%)	Pharmacy n(%)	Medicine n(%)	*p*-value
Gender							
Male	101(68.7)	2(5.1)	1(2.3)	3(3.6)	12(19)	83(42.3)	*p* < 0.001
Female	46(31.3)	37(94.9)	43(97.7)	80(96.4)	51(81)	113(57.7)	
Class							
Freshman	90(61.2)	23(59)	29(65.9)	52(62.7)	32(50.8)	104(53.1)	*p* > 0.05
Senior	57(38.8)	16(41)	15(34.1)	31(37.3)	31(49.2)	92(46.9)	
Residence situation							
Living with parents or guardians	90(62.9)	20(52.6)	21(48.8)	41(50.1)	29(49.2)	43(22.3)	*p* < 0.001
Living alone or in a fraternity/sorority	53(37.1)	18(47.4)	22(51.2)	40(49.9)	30(50.8)	150(77.7)	
Follower of a religion							
Yes	90(61.6)	32(82.1)	34(79.1)	66(80.5)	36(58.1)	122(62.2)	*p* < 0.05
No	56(3.4)	7(17.9)	9(20.9)	16(19.5)	26(41.9)	74(37.8)	
High school institution							
Public school	44(29.9)	13(34.2)	15(34.1)	28(34.2)	11(17.5)	64(32.7)	*p* >0.05
Technical school	38(25.9)	10(26.3)	10(22.7)	20(24.4)	15(23.8)	44(22.4)	*p* > 0.05
Private school	65(44.2)	15(39.5)	19(43.2)	34(41.4)	37(58.7)	88(44.9)	*p* < 0.05
Schooling of the parents							
Higher education	89(61)	18(46.2)	23(53.5)	41(50)	46(74.2)	152(77.6)	*p* < 0.001
High school	45(30.8)	17(43.6)	13(30.2)	30(36.6)	10(16.1)	38(30.8)	*p* < 0.05
Did not graduate from high school	12(8.2)	4(10.3)	7(16.3)	11(13.4)	6(9.7)	6(3.1)	*p* < 0.001*

Notes: *p*: comparison of all courses. * The analyses were compromised due to less than 5 answers. Because of the similarity between the data from nursing and pharmacy students, and the small number of students in those groups, the courses were grouped for the sake of analysis. **The percentages were calculated based on the number of students that answered the question.

### Sexual Background

Table 2 shows that although most women self-declared as heterosexuals, there was a significantly higher proportion of bisexuals among freshman students (13.2%) when compared to the seniors (4%) (*p* < 0.05). There was no difference in the proportion of heterosexuals, homosexuals and bisexuals among female and male students (*p* > 0.05). Only 63% of freshmen women reported having started sexual activity, and, among the seniors, 13.4% of women reported they were not yet sexually active. Among the freshman and senior male students, more than 89% reported they were sexually active. The proportion of sexually active men (89%) among the freshman males was significantly higher when compared with that of women (*p* < 0.001). The reported age of initiation of sexual activity was significantly lower among men when compared with women (*p* < 0.001). Women reported a higher rate of stable relationships than men (*p* < 0.05). We observed that women reported more visits to the gynecologist than men to the urologist (*p* < 0.001), and having or not health insurance was not associated with a higher rate of consultations. Condom use was reported by more than 90% of the freshman and senior male students and by only 61.8% of freshman female students and 57.3% of senior female students.

**Table 2:** Sexual background comparing gender and class

Answer	Freshman women n(%)	Senior women n(%)	*p*1	Freshman men n(%)	Senior men n(%)	*p*2	*p*3
Average age (standard deviation)							
	19.71(1.88)	22.87(2.50)		20.79(3.59)	23.44(2.53)		
Sexual orientation							
Heterossexual	133(83.6)	117(93.6)	*p* < 0.05	98(87.5)	70(86.4)	*p* > 0.05	*p* > 0.05
Homossexual	5(3.1)	3(2.4)	*p* > 0.05*	7(6.2)	8(9.9)	*p* > 0.05	*p* > 0.05
Bissexual	21(13.2)	5(4)	*p* < 0.05	7(6.2)	3(3.7)	*p* > 0.05*	*p* > 0.05*
If the student had ever gone to a gynecologist or urologist							
Yes	143(87.7)	120(94.5)	*p* < 0.05	30(26.5)	23(26.7)	*p* > 0.05	*p* < 0.001
No/don't remember	20(12.3)	7(5.5)	*p* < 0.05	83(73.5)	63(73.3)	*p* > 0.05	*p* < 0.001
Sexually active							
Yes	102(63)	110(86.6)	*p* < 0.001	102(89.5)	76(89.4)	*p* > 0.05	*p* < 0.001
No	60(37)	17(13.4)	*p* < 0.001	12(10.5)	9(10.6)	*p* > 0.05	*p* < 0.001
Age at first intercourse							
	17.12(1.61)	18(2.23)		16.67(1.52)	17.16(1.98)		*p* < 0.001
If engaged in a serious relationship							
Yes	69(42.9)	75(59.5)	*p* < 0.05	41(35.7)	41(48.2)	*p* > 0.05	*p* < 0.05
No	92(57.1)	51(40.5)	*p* < 0.05	74(64.3)	44(51.8)	*p* > 0.05	*p* < 0.05
Uses condom							
Yes	63(61.8)	63(57.3)	*p* > 0.05	100(98)	71(93.4)	*p* > 0.05	*p* < 0.001
No	39(38.2)	47(42.7)	*p* > 0.05	2(2)	5(6.6)	*p* > 0.05	*p* < 0.001*
If has life insurance							
Yes	114(71.2)	100(78.7)	*p* > 0.05	72(63.2)	68(79.1)	*p* < 0.05	*p* > 0.05
No	46(28.7)	27(21.3)	*p* > 0.05	42(36.8)	18(20.9)	*p* < 0.05	*p* > 0.05

Abbreviations: *p*1, comparing freshman and senior women; *p*2, comparing freshman and senior men; *p*3, comparing women and men.

Notes: *Analyses compromised due to less than 5 answers had been filled out in the questionnaire. **The percentages were calculated based on the number of people that answered the question.

### Knowledge about HPV and the Vaccine

Table 3 shows the proportion of correct responses to specific questions evaluating HPV-related knowledge. Of the 14 proposed questions, the proportion of senior female students answering correctly was significantly higher for 9 questions compared with their freshman counterparts, regardless of the undergraduate course. For men, in 12 out of the 14 questions, the proportion of senior students answering correctly was higher than that of their freshman counterparts. Comparing male and female students, regardless of class and course, in 6/14 questions the proportion of women answering correctly a question was higher than that of men. In general, Table 3 shows a clear progression of HPV-related knowledge throughout the years and a higher level of HPV-related education among women compared with men. For instance, the rate of knowledge that HPV is a sexually transmitted infection (STI) was high among freshman and senior female students (91.4% and 95.3% respectively) and among freshman and senior male students (82.3% and 94.2% respectively), being significantly lower among freshman male students (*p* < 0.05). The rate of knowledge that HPV can cause cervical cancer was of 82.2% and 85.8% among female freshmen and seniors respectively, and of 63.5% and 69.8% male freshmen and seniors respectively (*p* < 0.001). However, concerning the knowledge of other cancers related to HPV infection, such as vulvar, vaginal, anal, penile and oropharyngeal cancers, the accuracy rate was lower than 40% for both genders, with a significant increase in correct answers among senior students (*p* < 0.001). The percentage of students who knew that HPV can cause different types of warts was lower than the percentage of students who knew HPV could cause cervical cancer. However, this knowledge was higher among the senior compared with the freshman female students (62.7% and 37.4% respectively), and among the senior compared with the freshman male students (58.1% and 22.6% respectively).

**Table 3:** Knowledge about HPV comparing gender and class

Questions	Freshman women n(%)	Senior women n(%)	*p*1	Freshman men n(%)	Senior men n(%)	*p*2	*p*3
HPV is a sexually transmitted infection	149(91.4)	121(95.3)	*p* > 0.05	93(82.3)	81(94.2)	*p* < 0.05	*p* < 0.05
HPV is common	134(82.7)	109(85.8)	*p* > 0.05	68(59.6)	71(82.6)	*p* < 0.001	*p* < 0.001
Both genders can be affected by HPV	144(88.3)	115(90.6)	*p* > 0.05	95(83.3)	77(89.5)	*p* > 0.05	*p* > 0.05
The incidence of HPV is higher between 15 and 25 years old	115(70.6)	105(82.7)	*p* < 0.05	69(60)	63(73.3)	*p* < 0.05	*p* < 0.05
Most infections are asymptomatic	82(50.3)	100(78.7)	*p* < 0.001	40(34.8)	58(67.4)	*p* < 0.001	*p* < 0.05
The infection can become latent	101(62)	94(74)	*p* < 0.05	63(54.3)	63(73.3)	*p* < 0.05	*p* > 0.05
The infection can persist	121(74.2)	90(70.9)	*p* > 0.05	73(62.9)	66(76.7)	*p* < 0.05	*p* > 0.05
HPV can cause genital, anal and oropharyngeal warts	61(37.4)	79(62.7)	*p* < 0.001	26(22.6)	50(58.1)	*p* < 0.001	*p* < 0.05
HPV can cause cervical cancer	134(82.2)	109(85.8%)	*p* > 0.05	73(63.5)	60(69.8)	*p* > 0.05	*p* < 0.001
HPV can cause vaginal cancer	27(16.8)	46(36.5)	*p* < 0.001	11(9.6)	28(32.6)	*p* < 0.001	*p* > 0.05
HPV can cause anal and anorectal cancer	22(13.7)	45(35.7)	*p* < 0.001	9(7.9)	34(39.5)	*p* < 0.001	p > 0.05
HPV can cause penile cancer	17(10.6)	39(31)	*p* < 0.001	14(12.3)	32(37.2)	*p* < 0.001	*p* > 0.05
HPV can cause oropharyngeal cancer	15(9.3)	40(31.7)	*p* < 0.001	8(7)	25(29.1)	*p* < 0.001	*p* > 0.05
The types of HPV that cause warts are different from those that cause cancer	18(11)	54(42.5)	*p* < 0.001	9(7.8)	32(37.2)	*p* < 0.001	*p* > 0.05

Abbreviations: *p*1, comparing freshman and senior women; *p*2, comparing freshman and senior men; *p*3, comparing women and men.

Note: The percentages were calculated based on the number of people that answered the question.

**Fig. 1:**
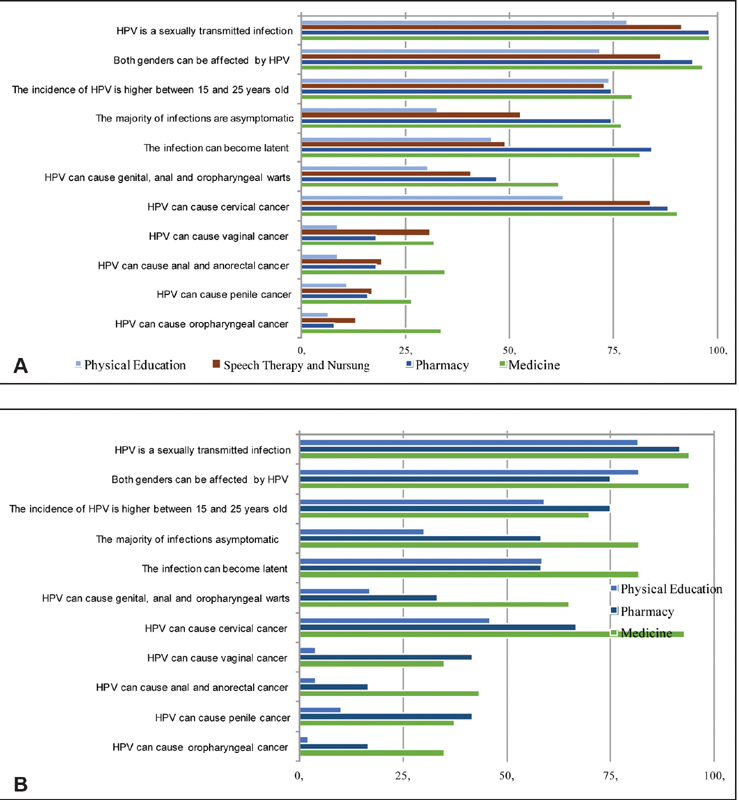
Knowledge about HPV comparing the percentage of right answers for the female (**A**) and male (**B**) students of the courses analyzed.

We observed a trend in general HPV-related knowledge among female and male students. Fig. 1A shows the comparison between the women of the different courses: we observed that physical education students know less about HPV infection and its consequences than those of speech therapy and nursing, who know less than pharmacy students, who, in turn, know less than medical students (*p*-trend < 0.001). The accuracy rate in the questions that stated that HPV is an STI that can affect both genders and can cause cervical cancer was higher than 60% among all of the women, and obeyed the proposed standard (*p*-trend < 0.001). On the other hand, knowledge that HPV has a higher prevalence among young adults aged 15 to 25 years did not show any difference between the courses (*p*-trend > 0.05). Knowledge that HPV infection is most often asymptomatic, that the infection may become latent and cause genital, anal and oropharyngeal warts showed a large difference among courses (*p*-trend < 0.001). Only 32.6% of physical education students knew that HPV can be asymptomatic, compared with 52.5% of speech therapy/nursing students, 74.5% of pharmacy students, and 77% of medicine students. As for the knowledge that warts can be caused by HPV, 30.4% of physical education students, 40.5% of speech therapy/nursing students, 47.1% of pharmacy students, and 61.9% of medicine students knew about this correlation. Regarding the other cancers related to HPV infection, the percentage of right answers among all of the women was below 40%. In the case of anal and anorectal cancer, the rate was of 8.7% of physical education students, 19.2% of speech therapy/ nursing students, 18% of pharmacy students, and 34.5% of medicine students (*p*-trend < 0.001). In the case of oropharyngeal cancer, 6.5% of physical education students, 12.8% of speech therapy/nursing students, 8% of pharmacy students, and 33.6% of medicine students recognized HPV as a cause (*p*-trend < 0.001). Regarding the relationship between infection and the development of penile cancer, the percentage of correct answers among women was of 10.9% of physical education students, 16.7% of speech therapy/nursing students, 16% of pharmacy students, and 26.5% of medicine students (*p*-trend < 0.05).

Fig. 1B shows the proportion of correct answers by male participants for the questions measuring HPV-related knowledge. Physical education students have less knowledge about HPV infection and its consequences than pharmacy students, who, in turn, score worse than medicine students (*p*-trend < 0.001). Due to the small number of male students in the nursing and speech therapy courses, the inclusion of them in the analyses was compromised. The percentage of correct answers regarding the sexually transmitted character of HPV infection was higher than 75% for all courses, with 81.6% for physical education, 91.7% for pharmacy and 94% for medicine (*p*-trend < 0.05). As for the knowledge that both genders can be affected and that the infection has a higher incidence among those aged between 15 and 25 years, the percentage of correct answers was of 81.8% and 59% respectively among physical education students, 75% and 75% respectively among pharmacy students, and 94% and 69.9% respectively among medicine students. Concerning the asymptomatic nature of the majority of HPV infections, physical education students obtained a rate of 30% of correct answers, while the rate for the pharmacy students was of 58.3%, and of 72.3% for the medicine students (*p*-trend < 0.001). Few male students knew that HPV causes genital, anal and oropharyngeal warts (17% of physical education, 33.3% of pharmacy and 65.1% of medicine students; *p*-trend < 0.001). For the question about the causal role of HPV in cervical cancer, the rate of correct answers ranged from 46% for the physical education students, and 66.7% for the pharmacy students, to 92.8% for the medical students (*p*-trend < 0.001). As of other cancers related to HPV, the rate of correct answers was low, with penile cancer being reported as a possible consequence of HPV infection by 10.1% of physical education students, 41.7% of pharmacy students, and 37.3% of those studying medicine.

### Attitude toward the Vaccine among Non-immunized Students and Intention to Indicate the Vaccine

Table 4 shows that the rate of women (26%) who had taken the HPV vaccine prior to the application of the first questionnaire was significantly higher than that of men (8%) (*p* < 0.001), regardless of the class and course. As for age at vaccination, there was a higher rate of freshman women who took the vaccine until age 17, and only 10 women took the vaccine until age 14 (data not shown). There was no difference in the proportion of girls vaccinated against HPV among the courses (data not shown). Non-vaccinated freshman women reported a greater interest in taking the vaccine (74.3%) than the seniors (61.4%) (*p* < 0.05). The intention to take the vaccine did not differ between women according to the course (data not shown). The intention to take the vaccine was significantly higher among unvaccinated women than among unvaccinated men (*p* < 0.05). Among men who did not take the vaccine, there was a higher proportion of pharmacy students that intended to take the vaccine compared with those of other courses (data not shown). Most men and women who answered the questionnaire would recommend the vaccine for both genders, and this proportion was higher among senior women (75.6%) than among freshman women (60.7%) (*p* < 0,05).

**Table 4:** Attitude toward taking the vaccine and to whom the students would recommend the vaccine comparing gender and class

Answers	Freshman women n(%)	Senior women n(%)	*p*1	Freshman men n(%)	Senior men n(%)	*p*2	*p*3
Vaccinated	44(27)	31(24.4)	*p* > 0.05	10(8.6)	7(8.1)	*p* < 0.05*	*p* < 0.001
Not vaccinated	104(63.8)	89(70.1)		80(69)	73(84.9)		
Did not know	15(9.2)	6(4.7)		23(19.8)	2(2.3)		
If not vaccinated (or doesn't know), would get vaccinated after answering this questionnaire							
Yes	78(74.3)	54(61.4)	*p* < 0.05	42(53.9)	46(61.3)	*p* > 0.05	*p* < 0.05
No	27(25.7)	34(38.6)		36(46.2)	29(38.7)		
Would recommend the vaccine							
To both genders	99(60.7)	96(75.6)	*p* < 0.05	76(65.5)	60(69.8)	*p* > 0.05	*p* > 0.05
Only to women	58(35.6)	27(21.3)	*p* < 0.05	30(25.9)	19(22.1)	*p* > 0.05	*p* > 0.05
Only to men	–	–		2(1.7)	2(2.3)	*p* > 0.05*	*p* < 0.05*

Abbreviations: *p*1, comparing freshman and senior women; *p*2, comparing freshman and senior men; *p*3, comparing women and men.

Notes: *Analyses compromised due to less than 5 answers had been filled out in the questionnaire. The percentages were calculated based on the number of people that answered the question.

### Vaccination against HPV before and after Filling out the First Questionnaire

Among the 233 students who answered the second questionnaire, which was applied 3 months after the first one, 39 women (28 medicine and 11 pharmacy students) and 15 men (14 medicine and 1 pharmacy student) had already taken the vaccine before the beginning of the research. Among the students who had filled out the first questionnaire and were not previously vaccinated, 34 women (30 medicine and 4 pharmacy students) and 10 male medicine students were eventually vaccinated against HPV. Concerning these two courses evaluated, there was an increase in the rate of vaccination. Between the 140 women that answered the second questionnaire, the rate of vaccination declared prior to the first questionnaire was of 28%, and it increased to 52% when we included women vaccinated in the three-month gap between questionnaires (*p* < 0.05). For men, the rate was of 16%, and it increased to 27% (*p* > 0.05).

## Discussion

After having applying the questionnaire to almost 500 Brazilian male and female university students, we observed that there was a greater knowledge related to HPV infection and its consequences among women, especially those in the medicine course. This knowledge was higher among senior students. In all courses and regarding both genders, the majority of the students were able to identify the sexually transmitted nature of the HPV infection, and they were aware that the prevalence of HPV infection peaks between the ages of 15 and 25 years. Although most students knew about the relationship between HPV infection and cervical cancer, most were unable to identify the association between HPV and other cancers such as vulvar, vaginal, anal, anorectal, penile, and oropharyngeal cancer. Likewise, few students knew about the relationship between genital, anal and oropharyngeal warts and HPV infection. Most students interviewed had not previously been vaccinated against HPV, and, even after being exposed to the correct answers to the questions, the vaccination rate increased only to 52% for women and 27% for men within the subsequent three months. It is interesting to report that during this period the vaccine was available at no cost to adults up to 26 years of age by the Brazilian Unified Health System (Sistema Único de Saúde, SUS, in Portuguese).

The importance of HPV education and its vaccine is to enable the public to make a conscious choice about vaccination, knowing the risks of HPV infection and vaccine protection.^8^ Similar to what was shown in our study, Balla et al^9^ observed that among high-school seniors in Hungary, few had sufficient knowledge regarding HPV infection: although most acknowledged its association with cervical cancer, their knowledge of other diseases was limited. Those authors also reported better knowledge on the part of female students. For Monteiro et al,^10^ the gender comparison showed a higher vaccination rate among women, which was similar to the findings made by Cooper et al^11^ that the knowledge and vaccination rate of men in higher education was low: only three quarters had prior knowledge of HPV, and just over half had knowledge about the vaccine. Because cervical cancer is a preventable disease and affects only women, national campaigns have been conducted for years in Brazil and worldwide, focusing on tracking cervical lesions and essentially educating women. Likewise, HPV vaccination was initially targeted at women in Brazil and worldwide, which also favored female education. We also found that 90% of the women interviewed had already gone to the gynecologist, which can be another cause of the higher knowledge.

In our study, the knowledge regarding almost all aspects related to HPV infection was higher among medical students, regardless of gender, when compared with nursing, speech therapy and pharmacy students, which is in line with the study by Chawla et al,^12^ who found greater knowledge about low-risk HPV and its relation to condylomas when comparing Indian gynecologists with paramedics. Yam et al^13^ also found greater knowledge among Hong Kong medical students in relation to other courses. In Brazil, Monteiro et al^10^ detected the same gap between literature and medicine students. The lack of knowledge related to HPV is even greater when considering courses not related to health care. In all of the questions in our questionnaire, physical education students performed poorly regardless of gender.

In our study, for both genders, senior students presented better knowledge when compared with freshman students. Similar data have been found in other studies^12^ ^14^ with university students, mainly in health care courses. This may be attributed not only to formal exposure to HPV-related information as part of the curriculum of the courses, but also to cumulative acquisition of HPV-related information from other sources of information, such as lay media. The majority (90%) of the students were aware of the sexually transmissible nature of HPV. This finding is in line with those from most of the studies performed with young university students globally.^6^ ^9^ ^14^ ^15^ Cervical cancer is unequivocally caused by high-oncogenic-risk HPV infection, and this type of cancer is the third most prevalent neoplasm and the fourth leading cause of cancer death among women.^3^ In our study, 76% of the participants were aware of the causal nature of HPV in cervical carcinogenesis. This question has been examined by several authors^5^ ^9^ ^14^ ^15^ ^16^ among students from a variety of courses, with the rate of correct answers ranging from 48 to 98%. Regarding the knowledge that HPV is related to other cancers, the proportion of correct answers did not exceed 40% for any type of cancer. This knowledge gap was found in other studies^9^ ^17^ for women and men. When we analyzed the relationship between HPV infection and the development of warts in our study and in the literature, knowledge was higher among higher-education students and among women.^15^ ^17^

According to our expectation, only a small proportion of students had been vaccinated against HPV: the rate of students evaluated in the first part of the survey was of 26% for women and of 8% for men, being higher among medicine students. Even within three months of the application of the questionnaire, the vaccination rate increased only to 52% for women and 27% for men. In Brazil, the quadrivalent vaccine was approved by the federal regulatory agency (Agência Nacional de Vigilância Sanitária, Anvisa, in Portuguese) in 2006, and it is recommended for adolescents and young adults. In the first years after the approval, the availability of the HPV vaccine was restricted to private clinics, and began to be offered by the SUS in 2014 for girls aged between 11 and 13 years. Since January 2017, its use has been extended to girls aged 9 to 14 years, boys aged 11 to 14 years, and men and women aged between 9 and 26 years who are HIV positive, transplanted or oncological patients on chemotherapy. Data from 2017 showed that vaccination coverage rate among girls aged 9 to 14 years in Brazil was of 82.6% for the first dose, and of 52.8% for the second dose. Among boys aged 12 to 13 years, the vaccination rate was of 43.8% for the first dose.^17^ In addition to not being easily accessible, concerns about the association with sexually transmitted infections (STIs), promiscuity, adverse effects, low recommendation of the vaccine by professionals, costs and lack of knowledge have led to the low rate of HPV vaccination in the target population.^13^ ^16^ Increased awareness had a positive effect on the intention to take the vaccine, and women were more likely to have a positive attitude toward vaccination.^9^ ^15^ Likewise, for Monteiro et al,^10^ more women were interested in taking the vaccine and, in general, higher-education students were more interested in the vaccination. In addition, only a third of the students had interest in taking the vaccine, and those were essentially the ones with the higher knowledge about HPV.

However, in the present study, data from the questionnaire applied three months after the first one showed an insufficient improvement in the proportion of vaccinated students. A study conducted in Scotland by McCusker et al^6^ evaluated the vaccination rate among medical students in 2008 and 2009. In 2008, no female students had taken all three doses of the HPV vaccine, and, in 2009, after an intense public campaign for HPV vaccination, 58% of the students had taken all three doses. For Attia et al,^17^ the vaccination rate will only grow significantly when the vaccine is administered in programs designed and organized to do so, such as school programs.

The help of governmental and non-governmental organizations working in the health sector is necessary to increase the knowledge on the part of the various health professionals and the general population regarding the various cancers and warts induced by HPV.^12^ Campaigns on the efficacy and safety of the vaccine are also required. The HPV vaccine is already proven to be responsible for the decrease in cases of cervical, oropharyngeal, penile, anal, vulvar and vaginal cancer. Likewise, a significant reduction in genital warts in vaccinated populations is observed. It is extremely important to increase the rate of vaccination among young people, especially before the beginning of sexual activity.^18^

The present study has some limitations. First, due to the fact that some of the courses had already finished the semester, the second questionnaire was only applied to students from two of the five courses that had filled out to the first questionnaire. Second, a homogenization of answers could have occurred by students comparing answers in class. The researchers reinforced the importance of individual answers to minimize this problem. Another limitation is that as the research included only students from health courses, we cannot conclude that the findings of our study represent all university students.

## Conclusion

So far, we conclude that knowledge and vaccination rates against HPV are still low among the university students evaluated in the present study. After we conducted the study, it became clear that higher knowledge is associated with a higher desire to get vaccinated. We hope that the results presented may lead to an investment in PHI programs, first to improve the vaccination rate, and, second, to guide the new university students about the risks of the infection and the methods to prevent it. The moment of starting university seems to be an open window of opportunity for campaigns of awareness once there is a gap in knowledge. It is also an opportunity to improve the rate of vaccination, as almost half of the freshman women declared being sexually inactive and would highly benefit from the vaccination, along with sexually active students who could be vaccinated in an attempt to catch up*.*

## References

[1] de Sanjosé S Brotons M Pavón M A The natural history of human papillomavirus infection Best Pract Res Clin Obstet Gynaecol. 2018 47 2 13. Doi: 10.1016/j.bpobgyn.2017.08.015 28964706 10.1016/j.bpobgyn.2017.08.015

[2] Castle P E Maza M Prophylactic HPV vaccination: past, present, and future Epidemiol Infect. 2016 144 03 449 468. Doi: 10.1017/S0950268815002198 26429676 10.1017/S0950268815002198

[3] Forman D de Martel C Lacey C J Soerjomataram I Lortet-Tieulent J Bruni L Global burden of human papillomavirus and related diseases Vaccine. 2012 30 05 F12 F23. Doi: 10.1016/j.vaccine.2012.07.055 23199955 10.1016/j.vaccine.2012.07.055

[4] Bruni L Diaz M Barrionuevo-Rosas L Herrero R Bray F Bosch F X Global estimates of human papillomavirus vaccination coverage by region and income level: a pooled analysis Lancet Glob Health. 2016 4 07 e453 e463. Doi: 10.1016/S2214-109X(16)30099-7 27340003 10.1016/S2214-109X(16)30099-7

[5] Nagpal J Linares L O Weiss J Schlecht N F Shankar V Braun-Courville D Knowledge about human papillomavirus and time to complete vaccination among vulnerable female youth J Pediatr. 2016 171 122 127. Doi: 10.1016/j.jpeds.2015.12.070 26846571 10.1016/j.jpeds.2015.12.070 PMC4808615

[6] McCusker S M Macqueen I Lough G Macdonald A I Campbell C Graham S V Gaps in detailed knowledge of human papillomavirus (HPV) and the HPV vaccine among medical students in Scotland BMC Public Health. 2013 13 264. Doi: 10.1186/1471-2458-13-264 23521847 10.1186/1471-2458-13-264 PMC3614879

[7] R Core Team. R: a language and environment for statistical computing [Internet] Vienna: R Foundation for Statistical Computing 2018 [cited 2018 Aug 10]. Available from: https://www.R-project.org/

[8] Ragin C C Edwards R P Jones J Thurman N E Hagan K L Jones E A Knowledge about human papillomavirus and the HPV vaccine–a survey of the general population Infect Agent Cancer. 2009 4 01 S10. Doi: 10.1186/1750-9378-4-S1-S10 19208201 10.1186/1750-9378-4-S1-S10 PMC2638455

[9] Balla B C Terebessy A Tóth E Balázs P Young Hungarian students' knowledge about HPV and their attitude toward HPV vaccination Vaccines (Basel). 2016 5 01 1 9. Doi: 10.3390/vaccines5010001 28036070 10.3390/vaccines5010001 PMC5371737

[10] Monteiro D LM Brollo L CS Souza T P Santos J RPD Santos G R Correa T Knowledge on the HPV vaccine among university students Rev Inst Med Trop São Paulo. 2018 60 e46. Doi: 10.1590/s1678-9946201860046 30231162 10.1590/S1678-9946201860046 PMC6169093

[11] Cooper D L Zellner-Lawrence T Mubasher M Banerjee A Hernandez N D Examining HPV awareness, sexual behavior, and intent to receive the HPV vaccine among racial/ethnic male college students 18–27 years Am J Men Health. 2018 12 06 1966 1975. Doi: 10.1177/1557988318803163 10.1177/1557988318803163 PMC6199446 30334489

[12] Chawla P C Chawla A K Shrivastava R Shrivastava A Chaudhary S Situation analysis of existing facilities for screening, treatment and prevention of cervical cancer in hospitals/primary health centers of Delhi-NCR region, India Asian Pac J Cancer Prev. 2014 15 13 5475 5482. Doi: 10.7314/apjcp.2014.15.13.5475 25041021 10.7314/apjcp.2014.15.13.5475

[13] Yam P WA Lam P L Chan T K Chau K W Hsu M L Lim Y M A cross sectional study on knowledge, attitude and practice related to human papillomavirus vaccination for cervical cancer prevention between medical and non-medical students in Hong Kong Asian Pac J Cancer Prev. 2017 18 06 1689 1695. Doi: 10.22034/APJCP.2017.18.6.1689 28670890 10.22034/APJCP.2017.18.6.1689 PMC6373786

[14] Jelastopulu E Fafliora E Plota A Babalis V Bartsokas C Poulas K Knowledge, behaviours and attitudes regarding HPV infection and its prevention in female students in West Greece Eur Rev Med Pharmacol Sci. 2016 20 12 2622 2629 27383314

[15] Chiang V C Wong H T Yeung P C Choi Y K Fok M S Mak O I Attitude, acceptability and knowledge of hpv vaccination among local university students in Hong Kong Int J Environ Res Public Health. 2016 13 05 486. Doi: 10.3390/ijerph13050486 27187424 10.3390/ijerph13050486 PMC4881111

[16] Sherman S M Bartholomew K Denison H J Patel H Moss E L Douwes J Knowledge, attitudes and awareness of the human papillomavirus among health professionals in New Zealand PLoS One. 2018 13 12 e0197648. Doi: 10.1371/journal.pone.0197648 30596646 10.1371/journal.pone.0197648 PMC6312361

[17] Attia A C Wolf J Núñez A E On surmounting the barriers to HPV vaccination: we can do better Ann Med. 2018 50 03 209 225. Doi: 10.1080/07853890.2018.1426875 29316825 10.1080/07853890.2018.1426875

[18] Ministério da Saúde. Secretaria de Vigilância em Saúde. Departamento de Vigilância das Doenças Transmissíveis. Coordenação-Geral do Programa Nacional de Imunizações [Internet]. Informe técnico da ampliação da oferta das vacinas papiloma vírus humano 6, 11, 16 e 18 (recombinante) – vacina HPV quadrivalente e meningocócica C (conjugada) Brasília, DF Ministério da Saúde 2018 [cited 2018 Dec 02]. Available from: http://portalarquivos2.saude.gov.br/images/pdf/2018/marco/14/Informe-T–cnico-HPV-MENINGITE.pdf

